# Wireworm feeding on potatoes during ripening is affected by soil moisture, tuber mass, and cultivar but not by tuber CO_2_ respiration

**DOI:** 10.1093/jee/toag012

**Published:** 2026-02-14

**Authors:** Michael Brunner, Michael Traugott

**Affiliations:** Applied Animal Ecology, Department of Zoology, University of Innsbruck, Innsbruck, Austria; Applied Animal Ecology, Department of Zoology, University of Innsbruck, Innsbruck, Austria

**Keywords:** integrated pest management, *Agriotes*, plant-insect interaction, irrigation, carbon dioxide

## Abstract

Wireworms, the larval stage of click beetles (Coleoptera: Elateridae), cause substantial damage to potato tubers, particularly during the ripening phase. Management strategies such as early harvest and the use of less susceptible cultivars have been shown to reduce feeding damage. However, the mechanisms driving cultivar susceptibility and the key factors influencing wireworm feeding during ripening remain poorly understood. To address this gap in knowledge, we compared the effects of harvest date, soil moisture, tuber mass, and wireworm abundance on feeding damage of wireworms (*Agriotes spp.*) during ripening in 2 potato cultivars. Additionally, we investigated whether differences in potato tuber CO_2_ respiration are responsible for cultivar susceptibility to wireworm damage. Feeding damage significantly varied between cultivars and harvest dates. Early harvest reduced damage in the highly susceptible cultivar but had no effect on the less susceptible cultivar. Soil moisture primarily affected wireworm abundance in the ridge, which decreased under low soil moisture conditions. In the susceptible cultivar, feeding marks increased significantly under low compared to high soil moisture. Tuber mass was positively related to damage, with heavier tubers showing more damage. Differences in tuber CO_2_ respiration between cultivars neither influenced wireworm feeding nor explained differences in susceptibility. These findings highlight the potential of selecting suitable cultivars and managing soil moisture, harvest timing, and tuber mass to reduce wireworm damage during potato tuber ripening. Understanding the mechanisms of cultivar resistance and breeding more resistant varieties will help minimize wireworm-related crop losses in potatoes.

## Graphical abstract

**Graphical Abstract:**
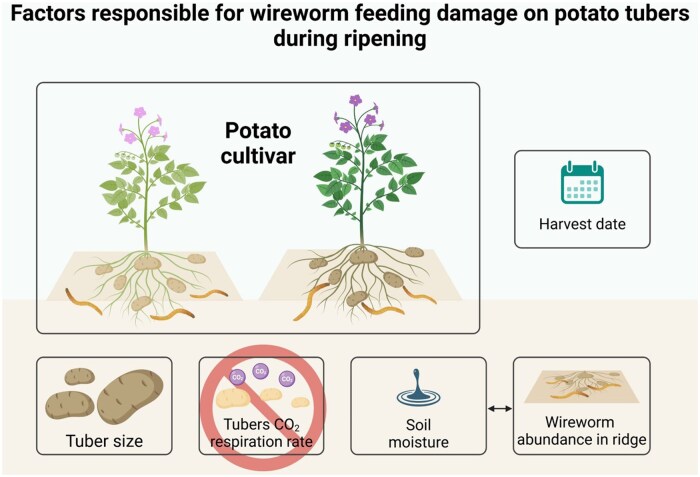

## Der Befall von Kartoffeln durch Drahtwürmer während der Reifungsphase wird durch die Bodenfeuchtigkeit, die Knollenmasse und die Sorte beeinflusst, nicht aber durch die CO_2_-Atmung der Knollen

Drahtwürmer, die Larven von Schnellkäfern (Coleoptera: Elateridae), verursachen insbesondere während der Abreife erhebliche Schäden an Kartoffelknollen. Eine frühe Ernte und der Einsatz weniger anfälliger Sorten können Fraßschäden reduzieren. Welche Mechanismen das Anfälligkeitsniveau einzelner Sorten bestimmen, sind bislang jedoch weitgehend unbekannt. Auch die wichtigsten Faktoren, welche den Fraß von Drahtwürmer während der Knollenreifung beeinflussen sind noch kaum erforscht. Um diese Wissenslücke zu schließen, wurden in dieser Studie die Auswirkungen von Erntedatum, Bodenfeuchte, Knollenmasse und Drahtwurmvorkommen auf die Fraßschäden durch Drahtwürmer (*Agriotes spp.*) während der Reifephase bei zwei Kartoffelsorten verglichen. Zusätzlich wurde untersucht, ob Unterschiede in der CO_2_-Atmung der Kartoffelknollen für die Anfälligkeit der Sorten gegenüber Drahtwurmschäden verantwortlich sind. Die Fraßschäden variierten erheblich zwischen den Sorten und Erntedaten. Eine frühe Ernte reduzierte die Schäden bei der sehr anfälligen Sorte, hatte jedoch keine Auswirkungen bei der weniger anfälligen Sorte. Die Bodenfeuchtigkeit beeinflusste in erster Linie die Anzahl von Drahtwürmern im Kartoffeldamm, wobei deren Anzahl bei geringer Bodenfeuchtigkeit abnahm. Gleichzeitig nahmen die Fraßspuren an den Knollen der anfälligen Sorte bei geringer Bodenfeuchtigkeit deutlich zu. Die Knollenmasse stand in einem positiven Zusammenhang mit den Schäden, wobei schwerere Knollen stärkere Fraßschäden aufwiesen. Unterschiede in der CO_2_-Atmung der Knollen zwischen den Sorten hatten keinen Einfluss auf deren Anfälligkeit für Drahtwurmschäden. Diese Ergebnisse unterstreichen das Potenzial von weniger anfälligen Sorten und zeigen, dass eine Steuerung der Bodenfeuchtigkeit und der Knollenmasse, sowie die Wahl eines frühen Erntezeitpunkts dazu beitragen können, Drahtwurmschäden während der Abreife von Kartoffelknollen zu reduzieren. Ein besseres Verständnis der Sortenresistenz sowie die Züchtung resistenterer Sorten können dazu beitragen, drahtwurmbedingte Ernteverluste bei Kartoffeln zu minimieren.

## Introduction

Wireworms, the larvae of click beetles (Coleoptera: Elateridae), inflict significant damage to a wide range of crops by feeding on below-ground plant parts. As a result, they are considered major soil-dwelling pests worldwide (Traugott et al. 2015, ­Vernon and van Herk 2022). Among root and tuber crops, potatoes are particularly affected because feeding marks reduce tuber quality and render them unmarketable (Fasulati et al. 2019). In addition, damaged tubers become more susceptible to secondary pathogens such as *Rhizoctonia solani*, once their skin—acting as a natural barrier—has been penetrated (Keiser et al. 2012).

The multiannual belowground lifestyle of wireworms complicates control efforts and limits the understanding of their ecology, making them a challenging pest to manage (Traugott et al. 2015, Nikoukar and Rashed 2022). Research on wireworm’s biology and ecology is crucial for developing novel management strategies (Poggi et al. 2021). In particular, understanding the foraging and feeding behavior on potato tubers is essential to mitigate severe damage during ripening (Chacon-Hurtado et al. 2023).

To locate host plants, wireworms generally follow a 3-step process: first, they orient toward carbon dioxide (CO_2_) emitted by belowground plant tissue, which triggers an unspecific search response, activating them to move from food-depleted to food-rich areas (Barsics et al. 2014, Traugott et al. 2015). Second, foraging is fine-tuned by specific root volatiles such as aldehyde compounds (Barsics et al. 2017, La Forgia et al. 2020). Third, once the wireworms have located the roots or tubers, they probe the plant tissue and then make a choice between acceptance or repulsion (Barsics et al. 2014). If the wireworm is repelled, it returns back to the CO_2_ source in order to orient toward an alternative food source (van Herk et al. 2015).

Despite several studies reporting differences in cultivar susceptibility (Olsson and Jonasson 1995, Kwon et al. 1999, Neuhoff et al. 2007, Johnson et al. 2008, Langdon and Abney 2017, Fasulati et al. 2019, Lorenzo et al. 2024), the mechanisms driving wireworm damage on potato tubers are still poorly understood. Carbohydrates, lipids, and proteins are expected to be responsible for an acceptance or rejection upon probing (Barsics et al. 2014, Johnson and Nielsen 2012), and feeding occurs preferably on tubers with higher monosaccharide contents (Crombie and Darrah 1947, Jonasson and Olsson 1994, Chacon-Hurtado et al. 2023). High levels of glycoalkaloids (α-solanine and α-chaconine), and chlorogenic acid, contribute to the resistance of some potato varieties to wireworm feeding damage but are likely not the primary factors driving their susceptibility (Jonasson and Olsson 1994, Johnson et al. 2008). Root volatiles have been shown to explain differences in wireworm damage between maize cultivars but these were not the only cues influencing feeding by wireworms (La Forgia et al. 2020).

Potatoes harvested from wireworm-infested soils early in the season have been proposed to be generally less severely injured than those harvested later (Hawkins 1937, Neuhoff et al. 2007). To avoid unfavorable soil conditions, wireworms burrow downwards, primarily during summer and winter ­(Traugott et al. 2015, Poggi et al. 2021). Therefore, soil temperature, moisture, and texture are important factors driving wireworm abundance in the upper soil layer and consequently their feeding damage (Nikoukar and Rashed 2022).

To better understand wireworm feeding behavior and differences in the susceptibility for wireworm feeding between potato cultivars during the ripening phase, we conducted a field experiment to test 3 hypotheses: (i) Wireworm feeding on potato tubers decreases with increasing soil moisture and increases under drier conditions. Under low soil moisture conditions, wireworms are more likely to feed on tubers in need of water. (ii) The CO_2_ respiration rate of potato tubers varies between cultivars. Cultivars with higher CO_2_ respiration rates suffer from increased wireworm feeding because CO_2_ serves as a cue for locating host plants. (iii) Wireworm damage to potato tubers will be lower at an early than at a late harvest, regardless of the potato cultivar. This is because an early harvest reduces the time tubers are exposed to wireworm feeding.

## Material and Methods

### Field Management

This study was conducted in an organic field at the research farm of the University of Innsbruck in Imst, Austria (N 47.221667, E 10.744361) in 2023. Based on previous studies, a consistently high density of 2 *Agriotes* species, *Agriotes ­sputator* (Linneatus, 1758) *(∼85%)* and *Agriotes obscurus (Linneatus, 1758) (∼15%)* was known to occur (Brunner et al. 2024). The field is located at 750 m above sea level, with a mean temperature of 6.5 °C and an average precipitation of 750 to 880 mm per year. The soil on the experimental field site contained 6% clay, 43% silt, and 51% sand at a pH of 7, and can be described as calcareous alluvial loamy sand. The experimental field, measuring 90 m × 27 m, was plowed in spring 2023 (20 April 2023) and prepared with a rotary harrow the following day. On 22 April 2023, potatoes were planted at a density of 2,500 kg per ha: cultivar *Solanum tuberosum* “Anuschka” (Cultivar A) in rows 5 to 32 and the cultivar *S. tuberosum* “Ditta” (Cultivar B) in rows 1 to 4 and 33 to 40 (Supplementary 1). Potato ridging was performed 3 times to control emerging weeds on 3 May 2023, 15 May 2023, and 12 June 2023. During the experimental phase, the field was neither irrigated nor were fertilizers or plant protection products applied. The 2 cultivars studied are firm-cooking table potatoes with smooth skins and shallow eyes. While “Anuschka” produces oval tubers and is characterized by a very early to early maturity, “Ditta” develops long-oval tubers and has a mid-early maturity. Cultivars were classified as “highly susceptible” (“Anuschka”) or “less susceptible” (“Ditta”) based on their observed response to wireworm damage in this study and prior observations (personal communication, C. Landzettel).

### Sampling Procedure and Soil Moisture Measurement

**Fig. 1.**
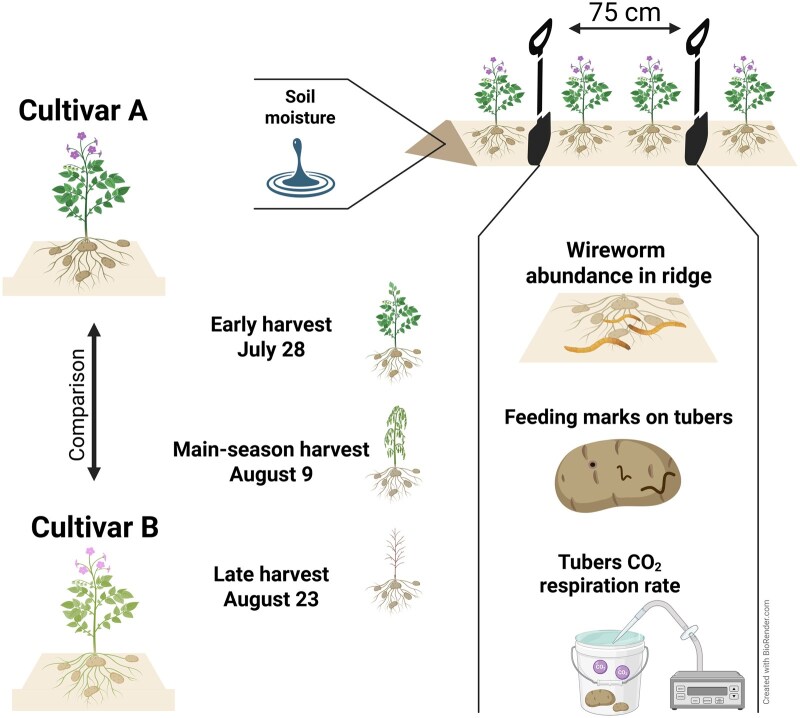
Study design and sampling scheme of this study. Potato tubers from 2 cultivars were harvested on 3 dates and examined for wireworm feeding damage. Additionally, wireworm abundance was measured for each soil sample. Tuber CO_2_ respiration was measured for each cultivar at the main-season harvest and the late harvest date.

Potato tubers for this study were hand-harvested where Cultivar A directly bordered a row of Cultivar B on 5 locations (replicates) for each cultivar and harvest date (Supplementary 1). A buffer zone of 3 m was maintained from the field margin, with sampling beginning beyond this zone. Three harvest dates were conducted in total: 28 July 2023 (Early harvest), 09 August 2023 (Main-season harvest), and 23 August 2023 (Late harvest). For each sample, 75 cm of the potato ridge was marked with 2 spades, and the potato tubers within this section were hand-harvested (Fig. 1). The soil from the 75 cm long part of the ridge was removed and searched for wireworms by sieving through a 6 mm mesh sieve (Outside Living Industries, Alkmaar, the Netherlands) to determine the wireworm abundance present in the ridge for each sample. Each sample was taken ∼40 cm down the row from the last harvest date. Soil temperature and soil moisture were continuously monitored at 30-min intervals throughout the experiment with a data logger (HOBO Data Loggers, Bourne, Massachusetts) with the probe placed at the center of the ridge.

**Fig. 2.**
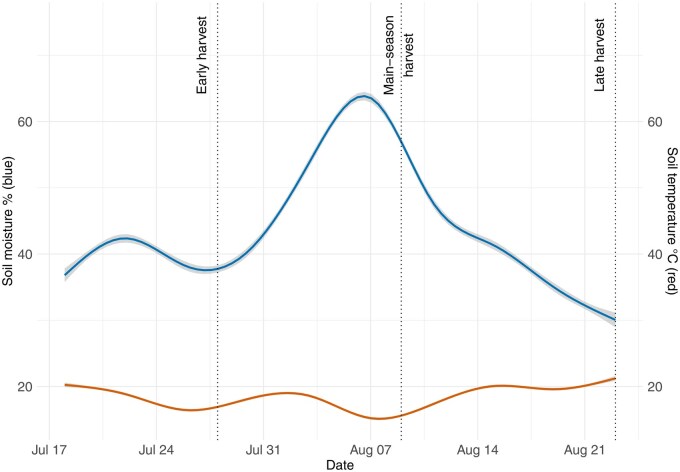
Soil moisture in % (blue line; left *y*-axis) and soil temperature °C (red line; right *y*-axis) during the experimental phase (*x*-axis) in the center of the potato ridge. The ribbon on the soil moisture line indicates differences for each day. The 3 harvest dates are indicated with a dotted line.

Soil temperature showed little fluctuation (±3 °C), averaging 18.4 °C during the experiment while soil moisture fluctuated strongly due to rain events. In the beginning, soil moisture remained relatively stable at approximately 40% (±4% variance) for 2 wk before the early harvest date (36.6%). Thereafter, soil moisture rose to 67.9% shortly before the main-season harvest date, before decreasing to 53.2% at the main-season harvest date and further dropping to 29.3% at the late harvest date (Fig. 2).

### Potato Tuber CO_2_ Respiration Rate and Wireworm Damage

Potato tuber CO_2_ respiration rates were recorded at the main-season and late harvest date but not at the first sampling due to technical issues. Therefore, harvested potato tubers were washed and gently brushed to remove all soil clinching to their surface. At both harvest dates, tubers were dried at room temperature for 30 min, and afterward, all tubers from 1 replicate were placed in a 5 liter plastic bucket (Bauhaus, Austria). The opening of the buckets was sealed with cling film (TOPPITS, MPreis, Austria). After allowing the tubers to rest in the sealed buckets for 2 h (Main-season harvest) or 3 h (Late Harvest), CO_2_ measurements were performed by inserting a 1,250 µl pipette tip (Biozym Scientific GmbH, Hessisch Oldendorf, Germany) through the cling film. The pipette tip was connected to an EGM-4 environmental gas monitor (PP Systems, Amesbury, Massachusetts) to measure CO_2_. For each replicate, the highest CO_2_ value recorded within 1 min of insertion was noted. Afterwards, the mass of each tuber was measured to account for size differences, and the CO_2_ respiration rate per surface area was calculated. To determine the surface area (SA) for each tuber, an average potato tuber density of 1.06 g per cm^3^ ­(Dalvand 2011) was assumed, the tuber was approximated as a sphere, with its size employed in the equation:


$$S=4\pi +{\left(\frac{3\times Weight}{4\pi \times Density}\right)}^{\frac{2}{3}}$$

To calculate the CO_2_ (ppm) respiration rate per unit surface area (cm^2^), the peak CO_2_ respiration of each replicate was divided by the total surface area of all tubers within 1 replicate. Respiration rate per unit surface area was then divided by the time potatoes rested in the sealed bucket to account for differences between harvest dates and get CO_2_ (ppm) respiration rate per unit surface area (cm^2^) and hour. After CO_2_ respiration rate measurement, potato tubers were evaluated for wireworm damage. Therefore, wireworm feeding marks per potato tuber were counted and other damage such as *Dry core (Rhizoctonia solani)*, scab coverage, and other feeding marks (for example mice, white grubs, or slugs) were recorded.

### Data Analysis

Data analysis was performed in R version 4.4.2 (R Core Team 2023). Data wrangling was performed with the package “dplyr” (Wickham et al. 2023) and plots were created with “ggplot2” (Wickham 2016). Wireworm abundance (wireworm count per replicate) was used as the response variable with soil moisture and harvest date as predictors. In addition, wireworm abundance was evaluated using harvest date and cultivar as predictors. Interaction terms were included when testing combined effects, such as between harvest date and cultivar or cultivar and wireworm abundance. Damage severity (number of feeding marks per tuber) was treated as the response variable, with harvest date, cultivar, and their interaction as predictors. Tubers with one or more feeding marks were classified as damaged, while tubers with no feeding marks were classified as undamaged. To predict the probability of damaged tubers, tuber damage (1 = damaged, 0 = undamaged) per tuber was used as the response variable, with harvest date, cultivar, and their interaction as predictors in a binomial generalized linear model. To assess the role of CO_2_ respiration in feeding severity, the number of wireworm feeding marks per tuber was treated as the response variable, with cultivar, log-transformed CO_2_ respiration per unit surface area, and their interaction as predictors. To assess factors influencing the probability of damaged tubers, tuber damage per tuber was treated as the response variable, with log-transformed potato tuber mass, wireworm count, cultivar, and the interaction between cultivar and wireworm count as predictors, assuming a binomial error distribution. To examine factors influencing damage severity, the number of wireworm feeding marks per tuber was treated as the response variable, with log-transformed potato tuber mass, wireworm count, cultivar, and the interaction between cultivar and wireworm count as predictors. All models, except for models in which tuber damage probability was assessed as the response variable, assumed a Poisson error distribution. All models used, and detailed test statistics are reported in the Supplementary Material (Supplementary 2).

To confirm that model assumptions were met, diagnostic plots for each model were examined according to Zuur et al. (2010). Post hoc pairwise comparisons were performed using estimated marginal means with Tukey adjustment for multiple testing with the “emmeans” package in R. To normalize data for regression models, potato mass and CO_2_ respiration were log-transformed. All statistical tests were performed at a significance level of 5% (alpha = 0.05). To generate a balanced dataset, tubers with less than 6.14 g (∼2.5 cm diameter) were excluded from data analysis. Due to high wireworm numbers, 1 plot in Cultivar A at the early harvest date was excluded from the analysis. If not stated differently, results are reported as mean ± SD.

## Results

### Wireworm Abundance

**Fig. 3.**
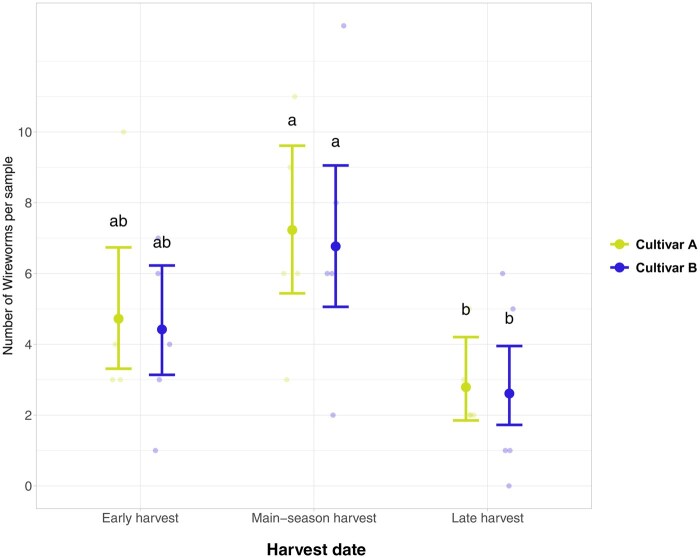
Wireworm abundance (y-axis) predicted with a generalized linear model for the 3 different harvest dates (*x*-axis) compared between the cultivars (color). Abundance was measured as the number of wireworm larvae per sample (*y*-axis ±SE). For each sample, wireworms in a 75 cm long section of the potato ridge were counted. Transparent points represent actual wireworm counts from this study. Data were analyzed using a generalized linear model (GLM) with a Poisson distribution. Post hoc pairwise comparisons were conducted using estimated marginal means with Tukey adjustment for multiple testing. Statistical differences at *P* < 0.05 are indicated by lowercase letters.

Wireworm abundance did not differ between potato cultivars (χ^2^ = 0.15; df = 1, 25; *P* = 0.7), but differed significantly between harvest dates (χ^2^ = 19.9; df = 2, 26; *P* < 0.01, Fig. 3). Wireworm abundance differed significantly between the main-season and the late harvest (*P* < 0.001) while the difference between the other harvest dates was marginally significant (*P* = 0.08; *P* = 0.09). Wireworm abundance increased significantly with higher soil moisture (χ^2^ = 501.75; df = 1, 549; *P* < 0.001).

### Wireworm Damage

#### Damage Probability

**Fig. 4.**
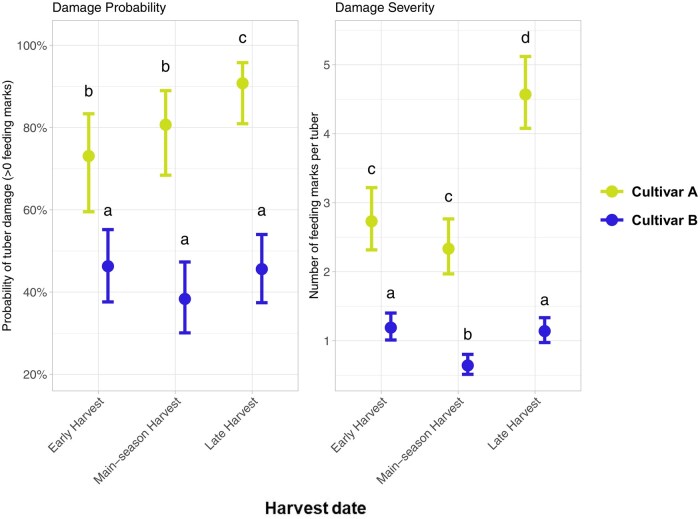
Wireworm damage on potato tubers compared between cultivars (color) and harvest dates using a generalized linear model. Damage probability (left panel) is based on the outcome of a binomial model, in which tubers with at least one feeding mark were classified as damaged, and is shown as predicted probability of damage (*y*-axis, ±SE) for the 3 harvest dates (*x*-axis). Damage severity (right panel) was assessed as overall wireworm feeding marks per tuber (*y*-axis, ± SE). Data were analyzed using a generalized linear model (GLM) with a Binomial distribution for damage probability and a Poisson distribution for damage severity. Post hoc pairwise comparisons were conducted using estimated marginal means with Tukey adjustment for multiple testing. Statistical differences at *P* < 0.05 are indicated by lowercase letters.

The probability of wireworm damage differed significantly between cultivars (χ^2^ = 73.8; df = 1, 611; *P* < 0.001), with Cultivar B showing lower overall probability of damage (43.5% ± 49.6%) than Cultivar A (82.2% ± 38.4%; Fig. 4). Harvest date alone did not significantly affect damage probability (χ^2^ = 3.5; df = 2, 612; *P* = 0.17). However, the interaction between cultivar and harvest date was marginally significant (χ^2^ = 5.0; df = 2, 609; *P* = 0.083), indicating cultivar-specific trends over time: For Cultivar A, the probability of damage increased over the season, with a significantly higher damage probability at the late harvest compared with both the early harvest (*P* = 0.05) and the main-season harvest (*P* = 0.04). In contrast, damage probability for Cultivar B did not differ significantly among harvest dates.

#### Damage Severity

Damage severity differed significantly between cultivars across harvest dates (χ^2^ = 309.4; df = 1, 611; *P* < 0.001), with Cultivar A exhibiting a higher number of feeding marks per tuber than Cultivar B (Fig. 4). Overall, Cultivar B showed consistently lower damage severity than Cultivar A.

Harvest date significantly affect damage severity (χ^2^ = 66.0; df = 2, 612; *P* < 0.001), and the interaction between cultivar and harvest date was significant (χ^2^ = 12.6; df = 2, 609; *P* = 0.002), indicating cultivar-specific trends over time: In Cultivar A, damage severity declined slightly from the early to the main-season harvest but increased strongly at the late harvest, which differed significantly from both earlier harvests (*P* < 0.0001). In contrast, Cultivar B exhibited low and relatively stable damage severity across harvest dates, with significantly lower damage at the main-season harvest compared with both the early and late harvests (*P* < 0.001).

### Tubers CO_2_ Respiration

The tubers’ CO_2_ respiration rate, measured for the main-season and late harvest date, varied significantly between potato cultivars (χ^2^ = 27,955.0; df = 1, 17; *P* < 0.001), with Cultivar B exhibiting higher respiration rates than Cultivar A at both harvest dates (Supplementary 3). Respiration rates also differed between harvest dates, with overall lower values at the late harvest compared to the main-season harvest (χ^2^ = 8,128.3; df = 1, 18; *P* = 0.02). However, the interaction between cultivar and harvest date was not significant (χ^2^ = 1,446.5; df = 1, 16; *P* = 0.32), indicating similar CO_2_ respiration rates of cultivars across harvest times.

**Fig. 5.**
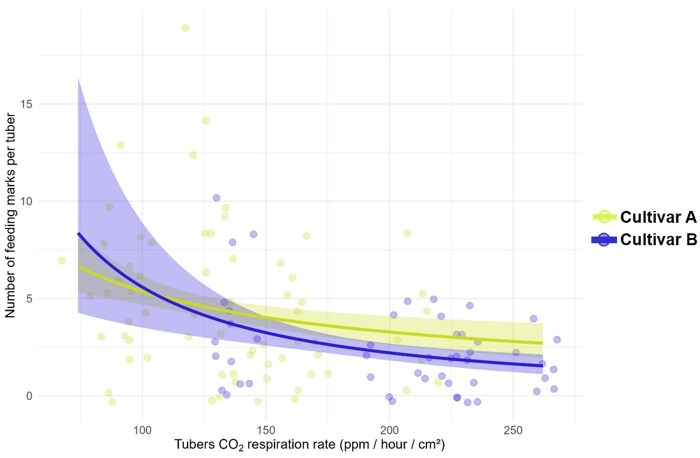
Generalized linear model showing the relationship between wireworm damage severity (*y*-axis) and tubers CO_2_ respiration (*x*-axis). Damage severity was measured as number of feeding marks per tuber and compared between potato cultivars (color). Points represent actual datapoints from this study. Data were analyzed using a generalized linear model (GLM) with a Poisson distribution.

Damage severity was significantly influenced by CO_2_ respiration rate (χ^2^ = 31.5; df = 1, 163; *P* < 0.001), with higher CO_2_ respiration rates being associated with fewer feeding marks (Fig. 5). However, the damage probability was not significantly affected by the CO_2_ respiration rate (χ^2^ = 0.18; df = 1, 35; *P* = 0.67, Supplementary 4).

### Tuber Mass and Number of Wireworms

**Fig. 6.**
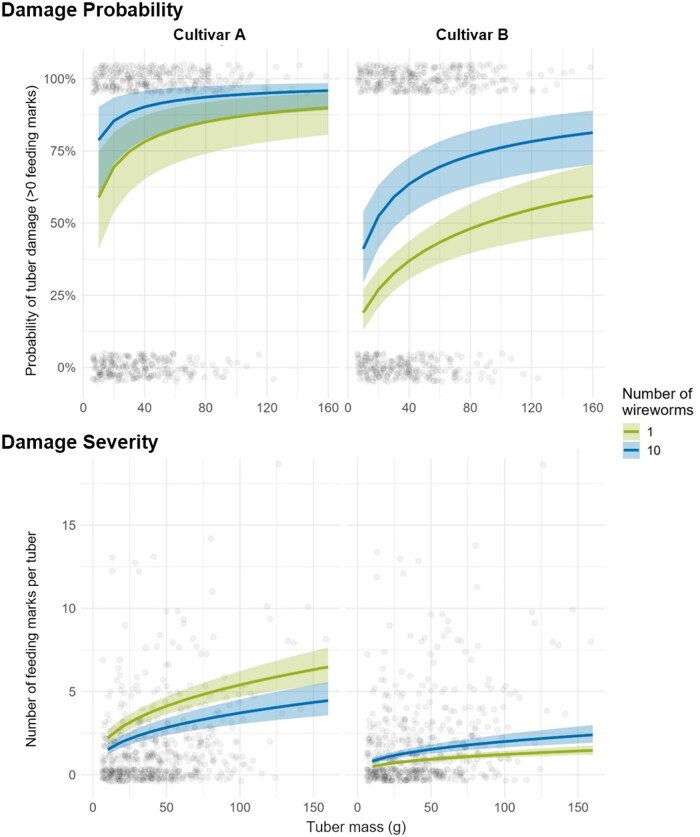
Generalized linear model showing the relationship between tuber mass (*x*-axis) and wireworm feeding damage at different wireworm abundances. Damage probability (top) is based on the outcome of a binomial model, in which tubers with at least one feeding mark were classified as damaged, and is shown as predicted probability of damage (*y*-axis), while the expected damage depending on the wireworm abundance is displayed in color. Damage severity (bottom) was assessed as the absolute number of feeding marks per tuber (*y*-axis). Transparent points represent actual data from this study. Data were analyzed using a generalized linear model (GLM) with a Binomial distribution for damage probability and a Poisson distribution for damage severity.

The potato tuber mass did not differ significantly among harvest dates or between cultivars (Supplementary 5). However, wireworm damage was strongly influenced by tuber mass. Heavier tubers had a higher number of feeding marks per tuber (χ^2^ = 199.28; df = 1, 1,100; *P* < 0.01; Fig. 6), and a higher probability of being damaged (χ^2^ = 43.1; df = 1, 1,100; *P* < 0.01).

Overall, damage severity was not significantly affected by wireworm abundance. However, this effect differed between cultivars, as indicated by a significant interaction (χ^2^ = 46.56, df = 1, 1,097; *P* < 0.01): In Cultivar A, damage severity slightly decreased with increasing wireworm abundance, whereas in Cultivar B, damage severity increased.

In contrast, the probability of tuber damage increased significantly with wireworm abundance, and this effect was similar for both cultivars (χ^2^ = 35.3, df = 1, *P* < 0.001). Cultivar identity also influenced tuber damage probability (χ^2^ = 154.1; df = 1, 1,099; *P* < 0.01), with consistent differences between cultivars independent of tuber mass and wireworm abundance.

## Discussion

**Fig. 7.**
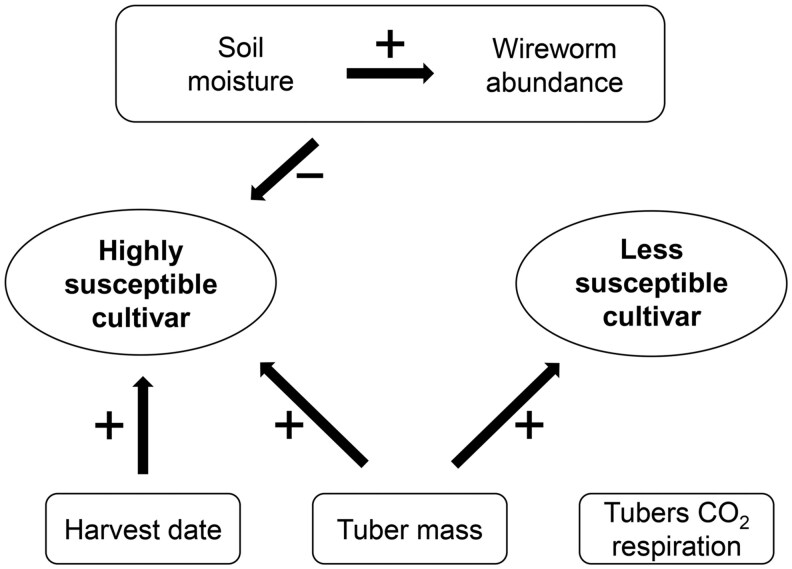
Main findings of this study showing factors affecting wireworm feeding damage during ripening in a highly susceptible cultivar (“Anuschka”) and a less susceptible cultivar (“Ditta”). Correlations are indicated with arrows and positive correlations to wireworm feeding damage are indicated with “+” and negative correlations with “−.”

Within this study, we identified potato cultivars, soil moisture, and tuber mass to be the main drivers of wireworm damage on potatoes during ripening. Soil moisture primarily influenced wireworm abundance in the ridge which increased with higher soil moisture. At a low soil moisture, feeding intensity on the highly susceptible cultivar increased, while it did not change on the less susceptible cultivar. With a late harvest date, the probability of wireworm damage increased for the highly susceptible cultivar but not for the less susceptible cultivar (Fig. 7).

### Soil Moisture and Wireworm Abundance

Firstly, we hypothesized that wireworm feeding on potato tubers decreases with increasing soil moisture and intensifies under drier conditions, as wireworms are more likely to feed on tubers to meet their water requirements. This hypothesis is supported for the highly susceptible cultivar by the results of this study, which show an increase in feeding intensity between the main season harvest (higher soil moisture) and the late harvest (lower soil moisture). In contrast, feeding intensity on the less susceptible cultivar did not change over the same period despite decreasing soil moisture.

However, soil moisture primarily affected the number of wireworms present in the ridge. The vertical distribution of wireworms is related to soil moisture with optimum conditions at 30% to 32% (Jung et al. 2014). Wireworms move deeper in the soil to avoid unfavorable soil moisture conditions (Parker and Howard 2001). We did observe similar movement patterns in our study, but wireworms were moving from the potato ridge to deeper soil layers already at higher soil moisture levels than previously observed. Despite low numbers of wireworms in the ridge at low soil moisture, the number of feeding marks on tubers increased. Feeding damage was therefore not positively correlated to the number of wireworms as was expected previously (Gibson 1956).

In wet conditions, wireworms have long been known to cause less damage to plants (Schaerffenberg 1942, Langenbuch 2009). Feeding on living plant tissues, such as potato tubers, can be stimulated by water deficiency, most likely to satisfy the need for water (Traugott et al. 2015, Samoylova and Tiunov 2017, Fasulati et al. 2019). Rapid changes in moisture levels in sandy soil have been proposed to trigger wireworms to burrow into tubers in search of water (Nikoukar and Rashed 2022). In our study, reduced soil moisture did not (Keiser et al., 2012) stimulate feeding in the less susceptible cultivar, as shown by the absence of an increase in damage between the main-season and late harvest, but in the highly susceptible cultivar. Here the reduction in soil moisture over the same period was associated with a strong increase in the number of feeding marks per tuber, while the proportion of damaged tubers remained stable. An explanation for the increased damage severity could be the location of tubers in the ridge. As soil moisture decreases, wireworms might tunnel into the tuber close by, and therefore tubers located deeper in the ridge might be affected more strongly. Alternatively, individual tubers of the highly susceptible cultivar might have a higher resistance than others. As a result, a few highly susceptible tubers receive repeated feeding marks which increases damage severity. More resistant tubers remain undamaged, leading to a stable overall damage probability. Unfortunately, these explanations remain speculative since the location of each tuber in the ridge was not recorded.

The effect of soil moisture on wireworm feeding damage has been shown to depend on the wireworm species (Langdon and Abney 2017). *Agriotes sputator*, the species most common in our experimental field, is a euryoecious species that is well adapted to a broad spectrum of environmental conditions (Staudacher et al. 2013). Because the effect of drying soil on movement and feeding behavior is species-specific, the observed behavior might not account for other species such as *A. ustulatus* or *A. brevis* which are more adapted to dry conditions and therefore respond less strongly to dry conditions (Furlan 1998, Nikoukar and Rashed 2022). Due to the importance of soil moisture for wireworms, water manipulation through irrigation has been recommended as a possible control approach for wireworms (Nikoukar and Rashed 2022).

Irrigation during, or after ripening to avoid wireworms feeding on potato tubers in search of water, has been applied by farmers with varying success. Our study indicates that irrigation during or after ripening would not effectively reduce the probability of wireworm feeding damage, particularly in susceptible cultivars. However, further research is needed to evaluate the potential of irrigation to reduce wireworm damage during potato ripening, as wireworm responses to changes in soil moisture are species-specific and may vary across different potato cultivars and wireworm species (Staudacher et al. 2013, Jung et al. 2014). Detailed knowledge is necessary to develop comprehensive recommendations for an optimal irrigation schedule that effectively reduces wireworm damage. At the same time, sustainable management considerations, such as avoiding excessive water use need to be taken into account.

### CO_2_ Respiration and Feeding Rate

Secondly, we hypothesized that the CO_2_ respiration rate of potato tubers varies between cultivars, and cultivars with a higher CO_2_ respiration rate were expected to experience increased wireworm feeding, because CO_2_ is known to be one of the most attractive compounds for soil-dwelling insect herbivores to locate host plants (Erb et al. 2013, Brandl et al. 2017). While we observed differences in CO_2_ respiration between cultivars, our results do not support this hypothesis, as tubers’ CO_2_ respiration rates did not influence the probability of wireworm damage. Moreover, as CO_2_ respiration rates increased, the number of feeding marks showed only a slight decrease, suggesting that differences in susceptibility to wireworm damage between potato cultivars are not driven by variations in tuber CO_2_ respiration.

Differences in the feeding preference of wireworms in 2 maize cultivars have been linked to the root emission of specific volatile organic compounds (VOCs) (La Forgia et al. 2020). However, besides VOCs, CO_2_ was proposed to affect the susceptibility through belowground concentration gradients and previous exposure to it (Doane et al. 1975, La Forgia et al. 2020). Chacon-Hurtado et al. (2023) found no differences in VOC emissions among potato tubers between cultivars. As a result, VOCs were deemed unlikely to drive differences in potato cultivars susceptibility. Instead other volatiles such as CO_2_ were expected to be responsible (Chacon-Hurtado et al. 2023). We did find differences in CO_2_ respiration between cultivars and even between harvest dates, but those had no effect on wireworm feeding damage during ripening. Tubers’ CO_2_ respiration rates are influenced by a multitude of abiotic and plant physiological factors ­(Dalvand 2011), which might have been the reason for the observed differences in CO_2_ respiration between harvest dates. In the soil, porosity and moisture can additionally affect the diffusion of CO_2_ (Barsics et al. 2013). Because tubers’ CO_2_ respiration changes constantly over time, and soil CO_2_ gradients vary with soil moisture, CO_2_ is likely relevant only for long-distance host location but not for fine-tuned host location over short distances (Arce et al. 2021). The role of CO_2_ emitted by potato tubers might therefore be less relevant for wireworm feeding on tubers, but for confirmation, season-wide experiments would be necessary.

### Harvest Date and Tuber Mass

Thirdly, we hypothesized that wireworm damage to potato tubers would be lower at an early harvest compared to a late harvest, regardless of the potato cultivar, because an early harvest reduces the time tubers are exposed to wireworm feeding (Hawkins 1937, Neuhoff et al. 2007). Our study confirms this hypothesis for wireworm damage probability in the highly susceptible cultivar, while neither damage probability nor damage severity increased over time in the less susceptible cultivar.

Wireworm damage was greater in heavier tubers than in lighter ones, an effect expected to be more pronounced at later harvests as tubers increase in size. Moreover, the increase in feeding marks with tuber mass was more pronounced in the highly susceptible cultivar. So far, the role of tuber mass in wireworm feeding damage has not been studied. There are 4 possible explanations for an increased feeding on heavier tubers: (i) Due to a larger surface area, wireworm feeding is more likely to occur on larger tubers. This would imply that feeding on potato tubers in a potato field happens at random without attractive substances such as VOCs or CO_2_ attracting wireworms. (ii) Larger tubers have taken a longer period to grow and were therefore exposed longer to wireworm feeding compared to smaller tubers. (iii) Larger tubers are more attractive due to the emission of a specific VOC blend or have higher monosaccharide contents, lower levels of glycoalkaloids (α-solanine and α-chaconine) or chlorogenic acid which all have been proposed to be associated with increased wireworm feeding on tubers (Jonasson and Olsson 1994, Chacon-Hurtado et al. 2023). (iv) Larger tubers are located deeper in the soil and are more exposed to vertically migrating wireworms. In contrast, tubers in the upper part of the ridge are less frequently reached, which results in increased feeding on the deeper, larger tubers. To mitigate wireworm damage on potatoes, cultivars with rapid tuber growth in combination with maturation control to avoid oversized tubers might be helpful to reduce wireworm feeding damage. Additionally, the role of tuber mass for wireworm feeding shown in this study underscores the need to account for tuber mass when evaluating novel control approaches or plant protection products. Otherwise, results could be misleading if the tuber mass was, for example, generally small or differs between treatment and control groups.

Overall, this study sheds light on wireworm feeding behavior and movement patterns in potato ridges during the harvest period. Planting less susceptible potato cultivars has substantial potential to mitigate wireworm feeding damage and ensure sustainable plant protection. Therefore, screening different cultivars for their susceptibility to wireworm damage is necessary to identify less susceptible cultivars. While tuber CO_2_ respiration was not found to be responsible for differences in potato cultivars’ susceptibility to wireworm damage, our findings emphasize the importance of potato cultivars, managing soil moisture, and harvest timing to reduce damage. Further research is needed to evaluate the impact of irrigation during ripening on wireworm density and feeding behavior as well as its effectiveness across different wireworm species. Additionally, the mechanisms which determine cultivar resistance need to be characterized to tailor breeding programmes toward less susceptible cultivars and develop effective control strategies.

## Supplementary Material

toag012_Supplementary_Data

## Data Availability

The datasets generated and/or analyzed during the current study are available at: https://osf.io/yhv6x/? view_only=34a34ff56ede429f9c8feb13e528edc7.
