# Supplementary material for: Wireworm feeding on potatoes during ripening is affected by soil moisture, tuber mass, and cultivar but not by tuber CO2 respiration
Source: J Econ Entomol. 2026 Feb 14;119(2):1024–34. doi: 10.1093/jee/toag012 (PMC13075814; doi:10.1093/jee/toag012)
Supplement: toag012_Supplementary_Data [file toag012_supplementary_data.docx]

**Wireworm feeding damage on potato tubers during ripening is driven by soil moisture, tuber mass, and cultivar but not by tuber CO_2_ respiration**

Michael Brunner^1^ and Michael Traugott^1^

^1^ Applied Animal Ecology, Department of Zoology, University of Innsbruck, Technikerstrasse 25, Innsbruck, Austria

Corresponding Author: Michael Brunner

Technikerstrasse 25, 6020 Innsbruck, Austria

michael.h.brunner@uibk.ac.at

T +43 (0)512 507-51892

Supplementary Material:


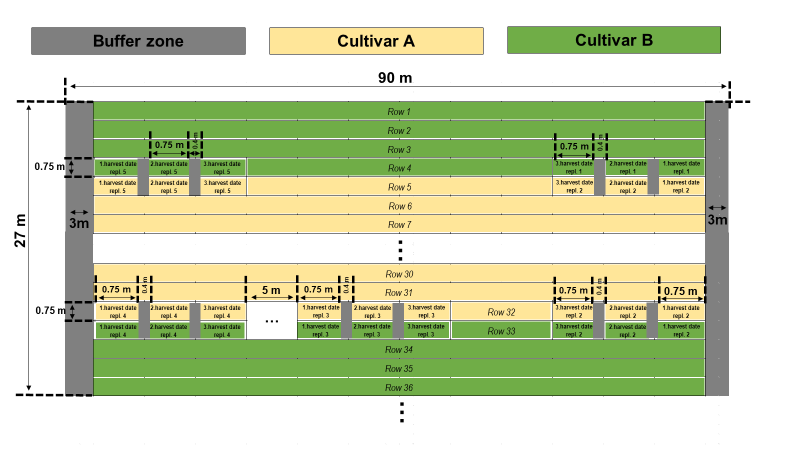


Supplementary 1: Schematic overview of the field experiment and the sampling.

Supplementary 2: *Overview of all models used to analyze the data of this study including the important test statistics.*

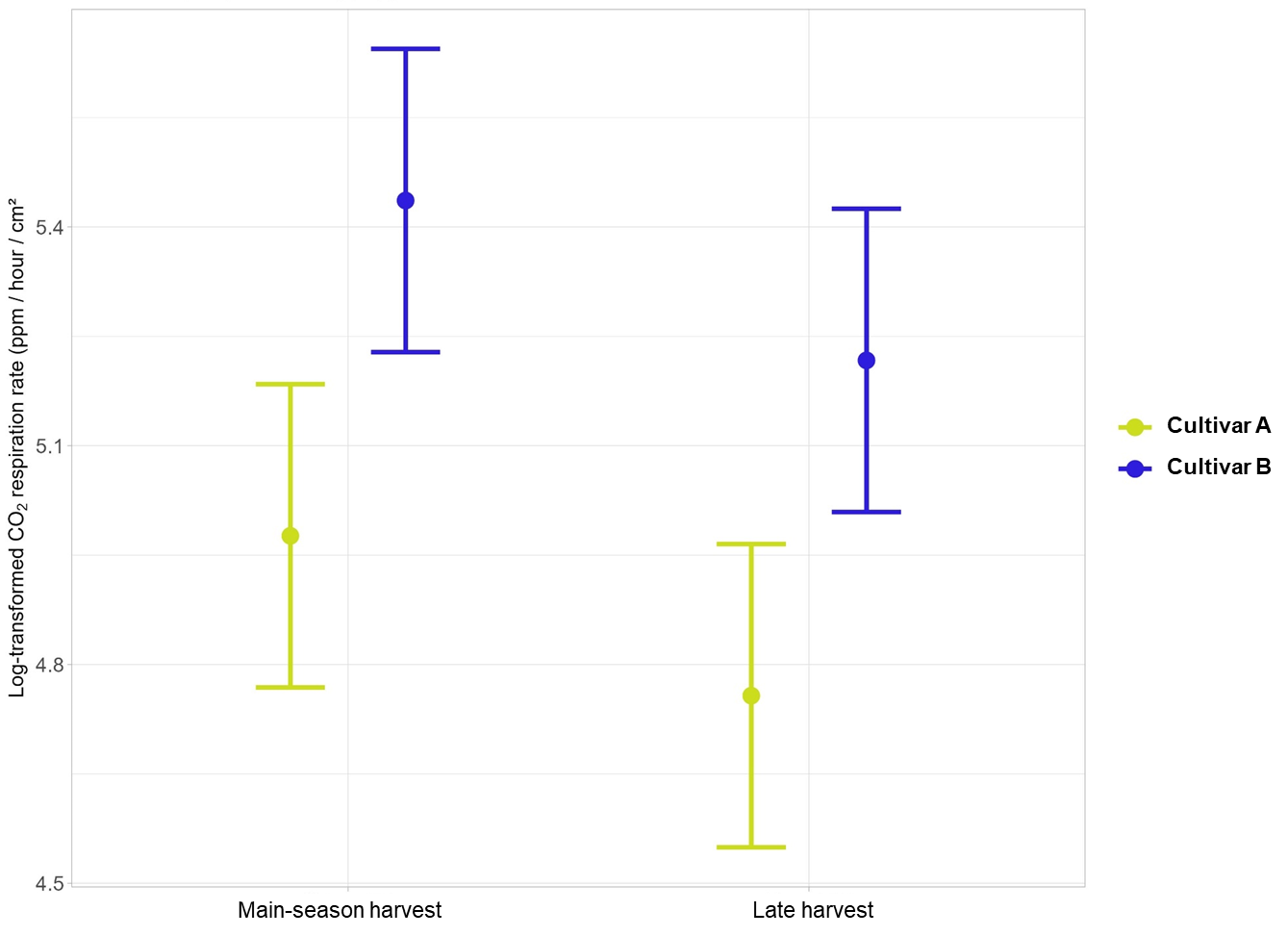


Supplementary 3: Potato tubers CO_2_ respiration rates (±SE) compared between cultivars (color) and harvest dates (x-axis) using a generalized linear model. CO_2_ respiration rates were calculated as ppm per hour and cm^2^ and log-transformed.


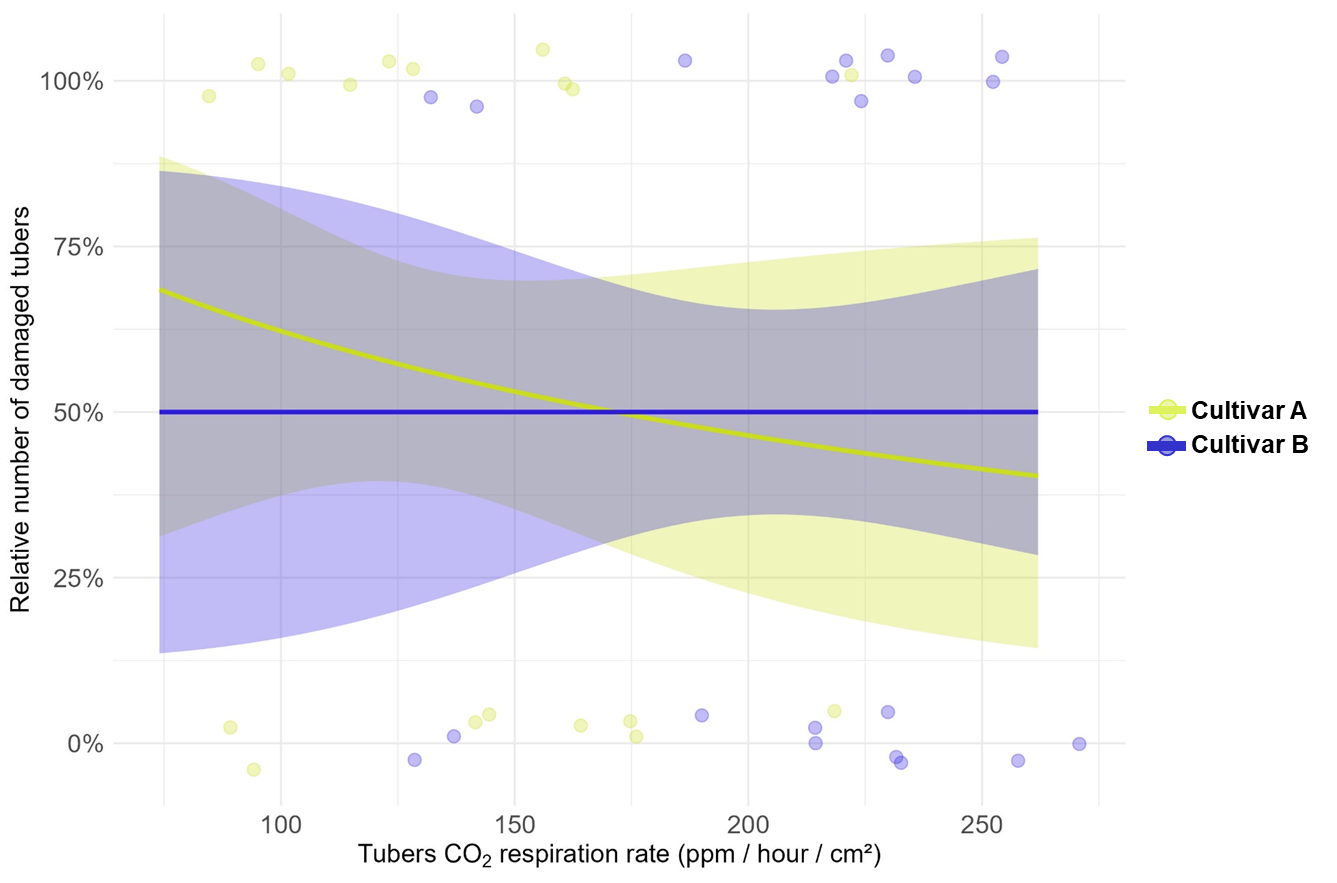


Supplementary 4: Generalized linear model showing the relationship between wireworm damage probability (y-axis) and tubers CO_2_ respiration (x-axis). Damage probability was measured as the relative number of tubers with one or more feeding marks and compared between potato cultivars (color). Points represent actual datapoints from this study.


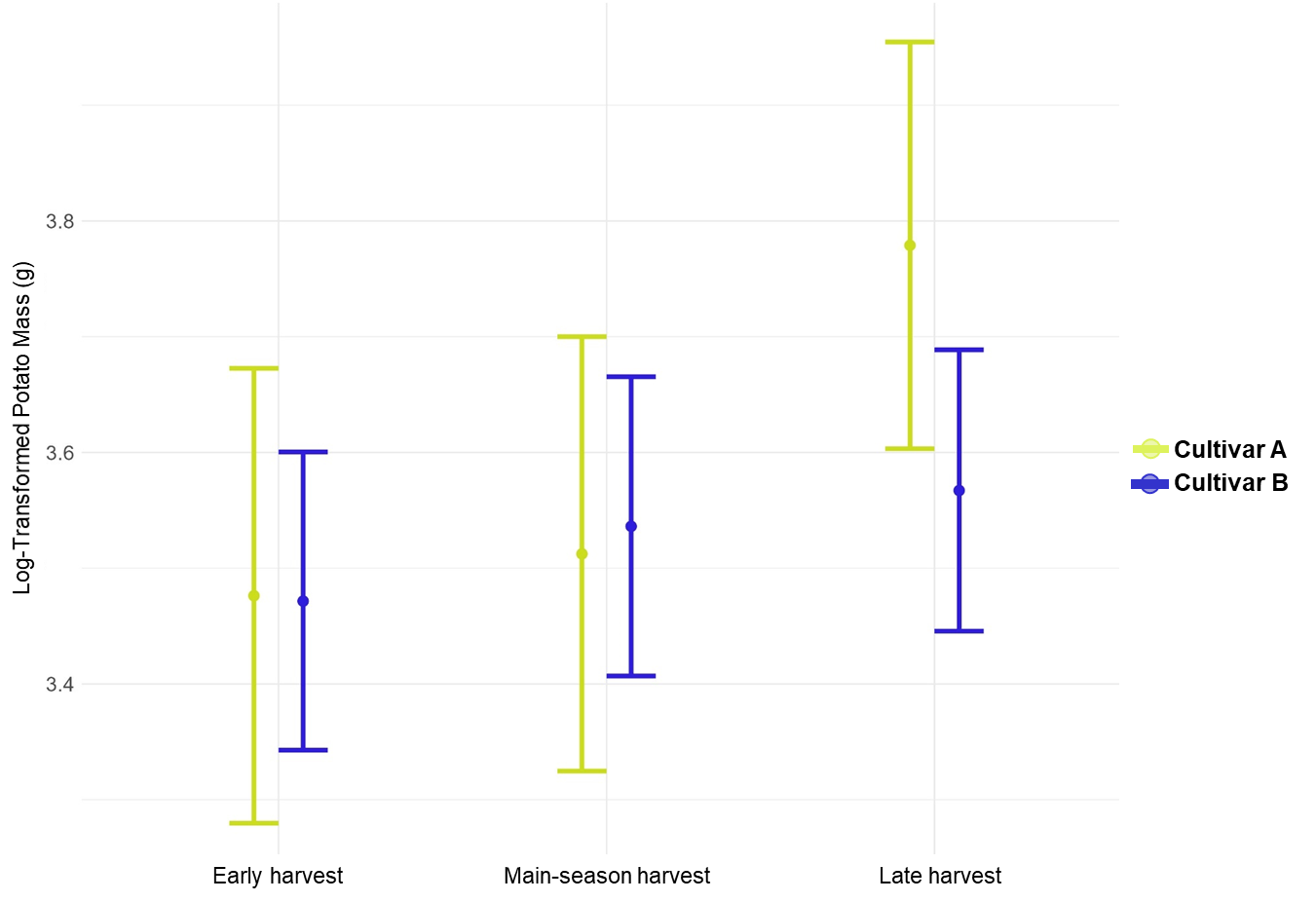


Supplementary 5: Potato mass compared between harvest dates (x-axis) and cultivars (color) using a generalized linear model. Potato mass (y-axis) was Log-transformed.
